# Physical and Metabolic Changes after Ileal Pouch-Anal Anastomosis: A Case Study

**DOI:** 10.3390/jfmk5040077

**Published:** 2020-10-28

**Authors:** Jacob Erickson, Patrick Harty, Paul Molling, Richie Stecker, Chad Kerksick, Andrew Jagim

**Affiliations:** 1 Sports Medicine, Mayo Clinic Health System, Onalaska, WI 54636, USA; erickson.jacob@mayo.edu (J.E.); molling.paul@mayo.edu (P.M.); 2 Energy Balance and Body Composition Laboratory, Texas Tech University, Lubbock, TX 79430, USA; patrick.Harty@ttu.edu; 3 Exercise & Performance Nutrition Lab, Lindenwood University, St. Charles, MO 63301, USA; rstecker@lindenwood.edu (R.S.); ckerksick@lindenwood.edu (C.K.)

**Keywords:** body composition, inflammatory bowel disease, J-pouch, physical activity levels

## Abstract

This case study examined changes in body composition, resting metabolic rate (RMR), aerobic capacity, and daily physical activity in a patient who had ulcerative colitis and underwent ileal pouch-anal anastomosis (IPAA) surgery. Body composition, RMR, and peak oxygen consumption (VO_2_peak) were assessed prior to surgery and four, eight, and 16 weeks after IPAA surgery. Daily physical activity data were extracted from a wrist-worn activity tracker preoperatively and 16 months postoperatively. At baseline, total body mass was 95.3 kg; body fat, 11.6%; lean body mass, 81.1 kg; RMR, 2416 kcal/d; and VO_2_peak, 42.7 mL/kg/min. All values decreased from baseline at four weeks postoperatively, body mass was 85.2 kg (−10.5%); body fat, 10.9% (−6.0%); lean body mass, 73.1 kg (−9.9%); RMR 2210 kcal/d (−8.5%) and VO_2_peak, 25.5 mL/kg/min (−40.3%). At 16 weeks postoperatively, most parameters were near their baseline levels (within 1–7%), exceptions were VO_2_peak, which was 20.4% below baseline, and RMR, which increased to nearly 20% above baseline. After the patient had IPAA surgery, his total and lean body masses, RMR, and aerobic capacity were markedly decreased. Daily physical activity decreased postoperatively and likely contributed to the decreased aerobic capacity, which may take longer to recover compared to body composition and RMR parameters.

## 1. Introduction

Inflammatory bowel disease (IBD), an autoimmune disorder, results in an inflammatory cascade that can lead to the destruction of the intestinal lumen. In 2015, IBD was diagnosed in an estimated 1.3% of adults in the US. The two subtypes of IBD are ulcerative colitis (UC) and Crohn’s disease. UC is characterized by severe inflammation in the large intestine that can cause diarrhea, bloody stools, abdominal pain, malaise, malnutrition, and weight loss [1,2,3]. Patients are often treated with corticosteroids or biologic therapies to mitigate inflammation and induce a state of remission. Additionally, a colectomy may be needed as a treatment of last resort, leaving patients with an ileostomy for fecal elimination [4].

To help patients regain a sense of normalcy and avoid permanent ileostomy, ileal pouch-anal anastomosis (IPAA), a procedure also known as J-pouch surgery, is commonly used in patients who have undergone a colectomy. The surgery involves removal of the entire colon and a portion of the rectum in combination with anastomosis of the ileal pouch to the anal canal with the placement of a temporary loop ileostomy [5,6]. The procedure is typically done as a three-step surgical process, which can be completed over several months or years. The second surgery is often considered the most invasive and physically challenging of the three, as this step requires the creation of the anastomosis (J-pouch) and the removal of any remaining colon and rectum [7].

It is likely that patients who opt for the three-step surgical procedure face physical stressors that present numerous challenges and inherent difficulties for maintaining routine physical activity and overall well-being. Patients with IBD also commonly have additional comorbidities and extraintestinal manifestations of IBD, which may create added challenges such as joint pain, skin issues, and fatigue [8]. Any major surgery and subsequent period of immobilization, particularly when coupled with dietary restrictions, may increase a patient’s risk for weight loss, reductions in lean body mass and decrease in peak performance.

Given the nature of each IPAA surgery, reductions in postoperative physical activity, and restrictions on lifting heavy objects, these alterations likely lead to marked reductions in musculoskeletal loading. These reductions may be magnified in patients who have relatively high states of fitness prior to surgery. However, because of the rarity of the condition and the challenges during the recovery process, a paucity of data is available on the specific physiologic changes that may occur throughout the IPAA surgical process and postoperative period in individuals with a high state of fitness. Therefore, the purpose of this case study was to examine the changes in metabolism, body composition, and performance that occur between the second and third operations of an IPAA three-step procedure.

## 2. Materials and Methods

### 2.1. Case Study

A 33-year-old Caucasian male gave consent for his clinical data to be used for this case study. The patient signed an informed consent document, which was approved by the Institutional Review Board of Lindenwood University (IRB# IRB-20-11) on 22 August 2019. The patient was diagnosed with UC, in 2014, which was 4 years before surgical intervention. He underwent a 3-step surgical procedure over the course of 19 months in 2017–2018, as a treatment option for managing UC and to create an IPAA. Formal data collection on the variables of interest did not begin until 5 days before the second of the 3 surgical procedures. The 4 time points of interest were baseline (T1), which was before the second operation, and 4 weeks (T2), 8 weeks (T3), and 16 weeks (T4) after the second operation. The patient was assessed for changes in body composition, resting metabolic rate (RMR), and peak oxygen consumption (VO_2_peak) during a maximal-effort graded exercise test on a treadmill. Historical data were collected from a fitness device worn on the patient’s wrist to document weekly changes in daily step counts. These data were recorded at T1 and up to 12 months after T4 (July 2018 to December 2019). 

### 2.2. Procedures

#### 2.2.1. Surgical Procedures

The patient underwent a subtotal colectomy with ileostomy placement 14 months before T1 as part of the 3-stage surgical procedure to treat the UC and create the IPAA to restore fecal transit. Shortly after T1, he underwent a second operation to create the IPAA and to remove the remaining rectal stump. The third stage of the surgical series to create the IPAA was completed in December 2018. Three months after the third stage, recurrent pouchitis was diagnosed. Additional complications included perianal fistulas and abscess formation, which led to further investigation and procedures that culminated in a new diagnosis of Crohn’s disease of the pouch. These complications led to re-diversion, placement of a new ileostomy, and 4 examinations under anesthesia for abscess and fistula management. These procedures were completed outside the primary testing window previously outlined. Given the complex course and associated morbidity, the patient opted for permanent ileostomy and injectable biologic therapy to manage Crohn’s disease of the pouch.

#### 2.2.2. Dietary Analysis

Before baseline testing (T1), the patient recorded his dietary intake over a 4-day period with an online commercially available nutrition-tracking program (MyFitnessPal; Under Armour Inc., Baltimore, MD, USA). Daily averages were calculated and presented as absolute and relative daily intake for energy, protein, carbohydrates, and fat.

#### 2.2.3. Anthropometrics, Body Composition & Bone Health

Total body mass and height were measured at each of the 4 time points of the study period. After the patient removed his shoes and excess clothing, his body mass was measured to the nearest 0.1 kg (Digital Scale BWB-627A Class III; Tanita, Arlington Heights, IL, USA), and his height was measured to the nearest 0.5 cm with a stadiometer (HR-200; Tanita, Arlington Heights, IL, USA).

Body composition was measured at each time point. The patient arrived at the laboratory 4 h after ingesting a standardized breakfast and abstaining from exercise and caffeine for 24 h. Measurements were made with dual-energy x-ray absorptiometry (DEXA) (Hologic Discovery A and Hologic APEX software version 4.5.3; Hologic Inc., Marlborough, MA, USA), the correction factor was from the National Health and Nutrition Examination Survey. 

#### 2.2.4. Resting Metabolic Rate (RMR)

After DEXA was completed, the patient lay supine on a padded examination table for assessment of RMR. A metabolic cart (TrueMax 2400 Metabolic Measurement System; ParvoMedics, Salt Lake City, UT, USA) was calibrated daily for less than 2% error. RMR (measured as kilocalories per day) was determined after 20 min of expiration as follows: (1) from data collected during the second 10 min, 5 consecutive minutes were identified in which the use of oxygen changed by less than 5%. (2) The mean of the oxygen use in each of those 5 min was calculated as the RMR.

#### 2.2.5. Peak Oxygen Consumption

The patient completed a graded exercise test on a motorized treadmill according to a modified Balke protocol [9]. During the test, the patient’s oxygen consumption was assessed with indirect calorimetry (TrueMax 2400 Metabolic Measurement System; ParvoMedics, Salt Lake City, UT, USA). The patient was instructed to maintain maximal effort across each graded stage until he reached volitional fatigue. VO_2_peak was then determined as the highest rate of oxygen consumption recorded during the test and expressed as VO_2_peak (in milliliters per kilogram per minute).

## 3. Results

Immediately before surgery, baseline data for the patient included the following: total body mass, 95.3 kg; body fat percentage, 11.6%; lean body mass, 81.1 kg; RMR, 2416 kcal/d; and VO_2_peak, 42.7 mL/kg/min. Baseline dietary intake is summarized in Table 1. 

When the baseline data were compared with the data from four weeks after surgery, the patient’s body mass had decreased to 85.2 kg (a decrease of 10.5%); body fat percentage was 10.9% (decrease of 6.0%); lean body mass was 73.1 kg (decrease of 9.9%); RMR 2210 kcal/d (decrease of 8.5%); and VO_2_peak was 25.5 mL/kg/min (decrease of 40.3%). 

**Figure 1:**
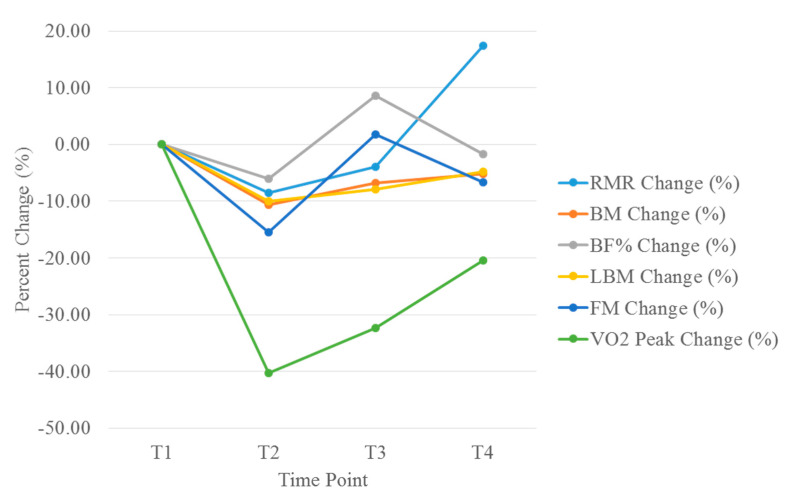
Changes in peak oxygen consumption (VO_2_peak), resting metabolic rate (RMR) and body composition parameters. VO_2_ = volume of oxygen consumption; RMR = resting metabolic rate.

**Figure 2:**
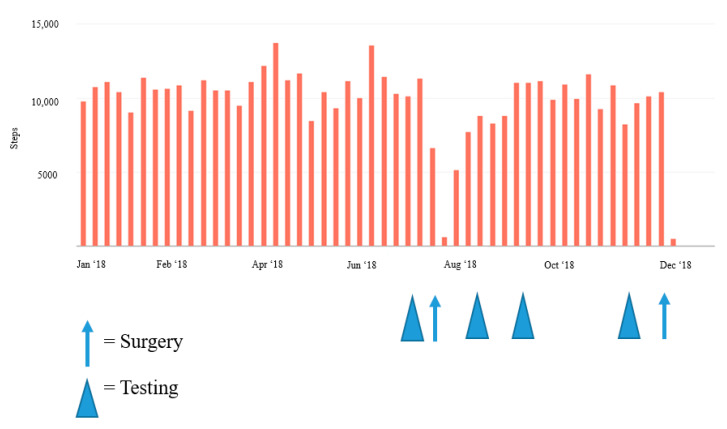
Weekly changes in step activity during 2018, pre- and post-operation.

**Figure 3:**
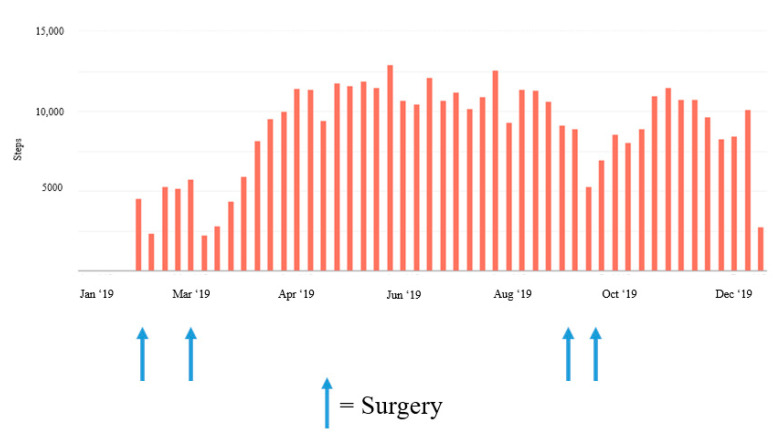
Weekly changes in step activity during 2019 pre- and post-operation.

Results at 16 weeks after surgery were close to the baseline results, with the exception of VO_2_peak, which was 34 mL/kg/min (20.4% less than the baseline value) and RMR, which increased to 17% above baseline values (Figure 1). Daily step activity decreased considerably (by 40–60%) in the weeks after each surgery (Figure 2 and Figure 3).

## 4. Discussion

To the best of our knowledge, this is the first study of its kind to examine postoperative changes in RMR, body composition, performance, and daily activity levels after an IPAA procedure in a patient with a high state of fitness. By four weeks after the second surgery (in July 2018), the patient’s body composition and metabolic parameters had decreased considerably (Figure 1), with the most notable reduction being a 40.3% decrease in aerobic capacity four weeks after surgery. This decrease in peak aerobic performance most likely resulted from cardiopulmonary deconditioning due to postoperative activity restrictions. Figure 2 shows a notable decrease in activity levels during the postoperative recovery period, which most likely further contributed to the deconditioned state and reduced aerobic performance. The increase in body composition parameters and RMR at eight weeks postoperatively coincided with the patient’s initiation of a light strength training program four to six weeks after surgery. Additionally, daily physical activity levels were also restored to near baseline levels around this time. However, aerobic capacity was still less than the baseline level at eight and 16 weeks postoperatively. Previous findings have indicated that patients who had undergone an IPAA procedure did not present with reduced workload capacity compared to reference norms when assessed at >15 months post-operation [10]. Therefore, it is possible that more time is required for a return to normal for peak aerobic performance.

After each surgical procedure, daily physical activity was considerably reduced and daily step counts were lower for several weeks (Figure 2 and Figure 3). Decreased physical activity in conjunction with the six weeks of postoperative lifting restrictions most likely contributed to the decrease in lean body mass measured at each postoperative time point. Likewise, as the patient returned to his regular physical activity, lean body mass increased to near baseline levels. In previous research, robust changes in body composition have also occurred after IPAA for UC management. Specifically, in 16 patients, mean fat-free mass decreased significantly two weeks after IPAA surgery, returned to baseline at three months postoperatively, and was higher than baseline at 12 months postoperatively [11]. Observational studies with longer durations have similarly noted long-term improvements in strength, total mass, lean body mass, fat mass, and bone mineral density after an IPAA procedure as patients recovered from the surgery itself and from the symptoms specific to active IBD [12]. Jensen et al. [12] reported an average increase in total tissue mass of 4.6 kg and in lean mass of 2.3 kg (compared with preoperative levels during the most active disease state) in a cohort of 20 patients with UC four to six years after IPAA surgery. It is worth noting that a recent study by Lan et al. [13] indicated that post-operative changes in body mass index of +10% or more may predispose patients to a greater risk of recurrent pouch sinus and therefore, post-operative nutritional and exercise strategies should focus on a slow return to baseline body weight with a specific emphasis on lean body mass accrual to avoid increases in body fatness.

An important consideration is that at baseline our patient had an above-average fitness level before surgery in July 2018, even after years of complications from UC. The body mass and lean body mass values of the current patient are well above those previously reported in the literature for UC patients and those who have undergone IPAA [11,12]. Jensen et al. [12] reported a mean body weight of 76 kg at four to six years after IPAA surgery. Similarly, a study by Christie and Hill [11] reported a mean body weight of 71 kg and a fat-free mass of 52 kg at 12 months after IPAA surgery—both values less than what was reported in the current case study.

Although they were assessed only at baseline, changes in dietary habits throughout the postoperative recovery period most likely influenced the magnitude of changes in body mass and composition. At baseline, the patient’s dietary habits were aligned with recommendations for active patients engaging in regular strength training activities (average self-reported intake, ~37 kcal/kg/day for energy and 1.84 g/kg/day for protein). Anecdotal reports from the patient described a severe decrease in appetite that began in the days immediately after the operation and lasted for two to four weeks. Additionally, the patient began a restricted diet in accordance with dietary recommendations after colorectal surgery and in alignment with ileostomy-specific guidelines. As evidenced by the robust reductions in total and lean body masses, it is likely that the postoperative total energy and protein intake were much lower than those reported at baseline. These reductions in body mass occurred despite reductions in daily physical activity; therefore, energy intake was likely still less than the reduced energy requirement, resulting in an overall negative energy balance. 

Surgery of any kind can lead to postoperative restrictions and may require modifications to certain exercises or physical activities. Anecdotal reports from our patient described the midline abdominal incision as the biggest contributor to the reduction in physical activity. Even well after the six-week lifting restriction had passed, the midline incision created challenges for recreational activities and certain exercises, particularly for any strength-based movement requiring a high degree of core engagement or abdominal bracing. The patient slowly increased daily activity levels as tolerated, with an early emphasis on walking (Figure 2 and Figure 3). The patient also resumed light strength training activities four weeks postoperatively, while still following the 4.5-kg lifting restriction. Most of these activities were body weight exercises with light free weights and resistance bands, which he performed on three or four days per week. At six weeks postoperatively, the patient initiated a light progressive strength training program using a combination of body weight exercises, stationary machines, and free weights, 3–4 days per week with the addition of blood flow restriction training for select exercises, in an attempt to provide an adequate training stimulus while reducing the required load. The patient completed 8 to 10 exercises per session, using 3–4 sets of 8 to 20 repetitions per set with an emphasis on maximizing muscle hypertrophy at a self-reported rating of perceived exertion level of four to six. These efforts appeared to help recondition the patient and promote increases in lean body mass to near baseline levels over time. Gradually, the patient was also able to return to normal daily physical activity levels as evidenced by the slow increase in daily step counts after surgery and the self-reported increased frequency in strength training sessions. However, VO_2_peak levels had not fully recovered at 16 weeks after surgery. Although they did not occur in the primary time frame of the study, the reductions in daily steps in early 2019 (Figure 3) are reflective of how pouchitis can also affect daily physical activity. The patient had several complications, which resulted in various symptoms that included incontinence, sleep disruption, fatigue, increased inflammatory markers, fever, and malaise, all of which likely contributed to the reductions in physical activity and reduced overall well-being. Postoperative complications of this nature are fairly common after this procedure, and previous research has indicated that nearly 30% to 45% of patients have postoperative perianal complications, such as incontinence, high frequency of output, abscesses, fistulas [7,14] and pouchitis (in up to 50% of patients) [7].

A person with lower baseline fitness and lean body mass may not expect the same magnitude of change over a period similar to that of our case study. This is important, however, because the majority of patients with IBD are in their mid to late thirties at the time of diagnosis and therefore may be challenged with maintaining regular physical activity, body composition, and physical fitness for several decades while living with IBD [15]. IBD patients commonly report multiple barriers to exercise, such as abdominal or joint pain, fatigue, disease flare-ups, urgency, loose stool, incontinence, and frequent bowel movements, all of which may hinder their ability to meet desired activity levels [16,17]. When regular exercise is possible, however, it has been shown to mitigate some of the deleterious effects of the disease and improve the overall quality of life for patients with IBD, who often describe exercise as an effective coping strategy [18]. 

Maintaining a high fitness level and greater amounts of lean body mass may even create a degree of resilience during disease flare-ups or surgical procedures. For example, higher preoperative levels of lean body mass have been associated with fewer complications and better outcomes among patients with Crohn’s disease who undergo surgery, particularly when they are malnourished [19,20]. There is also evidence that systemic inflammation occurs less in persons who are physically active and lean [21]. However, more research is needed to determine the specific effects of a higher fitness state on morbidity in patients with IBD who are undergoing surgery and for long-term management of the disease. 

## 5. Conclusions

Results of this case study provide evidence for potential changes in metabolism, body composition, performance, and daily activity that may occur when an active patient with IBD undergoes an IPAA procedure. Considerable reductions in all parameters occurred four weeks postoperatively, with total body mass and lean body mass returning to nearly baseline levels (within about 5%) at 16 weeks postoperatively after the implementation of a conservative and progressive strength training program as tolerated in conjunction with the restoration of daily physical activity levels. Daily physical activities may not return to normal levels until four to five weeks postoperatively, and aerobic performance may not fully return to baseline levels until more than 16 weeks postoperatively. Clinicians and sports medicine professionals can use this information as they guide patients through the recovery process after an IPAA procedure and advise them about expectations for potential postoperative changes in metabolism, body composition, performance, and activity level. These findings can also help direct future research in this area to examine the body composition and performance changes following colorectal surgery of this nature or to study the effects of perioperative nutrition and rehabilitative strategies. Additionally, this case study is the first to propose the use of low-load strength training in conjunction with blood flow restriction as a countermeasure to attenuate reductions in lean body mass after colorectal surgery.

## Figures and Tables

**Table 1:** Dietary intake at baseline.

Dietary Component	Absolute	Relative to Body Weight
Energy, kcal/day	3516	36.6
Protein, g/day	177	1.8
Carbohydrate, g/day	401	4.2
Fat, g/day	144	1.5

kcal/day = kilocalories per day; g/day = grams per day.
